# Preliminary Evaluation of a Large Language Model–Powered Chatbot for Osteoporosis Self-Management Education: Formative Randomized Controlled Trial

**DOI:** 10.2196/85475

**Published:** 2026-06-02

**Authors:** Jinling Huang, Xiaolian Xin, Chunyan He, Haoyan Xiong, Wenjun Pang, Shaobo Pang, Wei Sun, Xianghua Ding

**Affiliations:** 1 Affiliated Hospital of Guangdong Medical University Zhanjiang China; 2 University of Glasgow Glasgow, Scotland United Kingdom

**Keywords:** chatbots, large language models, self-management education, osteoporosis, adherence, patient education

## Abstract

**Background:**

With the increasing burden of chronic diseases, self-management education (SME) is crucial. Traditional SME based on face-to-face delivery by clinicians is resource-intensive, and general digital tools such as web-based platforms often provide limited interactivity for patient learning. Although chatbots based on large language models (LLMs) show promise in interactivity, their real-world effectiveness lacks empirical evidence.

**Objective:**

This study aimed to explore the feasibility and preliminary effectiveness of an LLM-based chatbot specifically designed for osteoporosis SME.

**Methods:**

A formative randomized controlled trial was conducted in a tertiary hospital from February 2024 to March 2025. Adults aged ≥18 years with osteoporosis were recruited and randomly assigned (1:1) to either the intervention (OPBot) group or a control group receiving traditional health education. The chatbot provided interactive educational content and question-and-answer support, while the control group received face-to-face education and written materials. Osteoporosis knowledge was assessed using the Osteoporosis Knowledge Assessment Tool at baseline and discharge. Nurses’ time spent on health education was self-recorded during each intervention session and aggregated across sessions. Adherence to disease management was assessed at 1, 3, and 6 months after discharge via telephone using Likert-scale questionnaires. The reliability of OPBot responses was evaluated by 2 clinician assessors using a 5-point Likert scale, with interrater agreement calculated using Cohen *κ*. Group comparisons were conducted using 2-tailed independent *t* tests, Mann-Whitney *U* tests, and chi-square tests, and adherence outcomes were analyzed using mixed-effects models.

**Results:**

A total of 100 participants were randomized; 12% (12/100) were excluded due to loss to follow-up, refusal of the second knowledge assessment, or death, leaving 88% (88/100) participants for analysis (n=45, 51.1% in the OPBot group and n=43, 48.9% in the control group). The OPBot group showed significantly higher postintervention knowledge scores than the control group (median 80.0, IQR 70.0-89.0 vs median 75.0, IQR 65.5-80.0; *P*=.01). Nurses in the OPBot group spent lesser time on SME than those in the control group (median 5.0, IQR 2.0-17.0 vs median 23.0, IQR 20.0-25.0 minutes; *P*<.001). For adherence outcomes, a significant group×time interaction was observed for calcium supplement intake (odds ratio 1.49, 95% CI 1.08-2.06; *P*=.02), indicating differing adherence trajectories over time. The OPBot group also showed higher odds of consuming calcium-rich foods across time points (odds ratio 2.87, 95% CI 1.04-7.89; nominal *P*=.04), although this association did not remain significant after Holm correction. No significant effects were observed for sun exposure (*P*=.56), exercise (*P*=.79), or total adherence scores (*P*=.33). In the question-and-answer module, most OPBot responses were rated as highly reliable 89.4% (76/85), with high interrater agreement (Cohen *κ*=0.83).

**Conclusions:**

LLM-based chatbots specifically designed for osteoporosis SME may improve patient knowledge, supporting adherence behaviors, and reducing healthcare workload. However, further large-scale studies are needed to confirm these findings.

## Introduction

The global prevalence of chronic conditions is an increasing concern. In 2024, the World Health Organization reported that chronic conditions accounted for more than two-thirds of deaths worldwide [1]. In response to increasing pressure on health care services, self-management plays a crucial role in addressing these conditions, involving an individual’s ability to manage symptoms, treatments, physical and psychosocial impacts, and necessary lifestyle changes [2]. Self-management education (SME) equips individuals with the essential knowledge and skills to manage their conditions effectively [3], making it an increasingly vital component of health care [4]. Without adequate education, individuals may struggle to understand their condition and lack management skills [5].

Traditionally, SME has been delivered by health care staff in medical settings such as hospitals or clinics, primarily through face-to-face lectures [6]. However, this approach is time-intensive and places a considerable burden on health care professionals. This burden is further exacerbated by ongoing workforce shortages and increasing service demands in modern health care systems [7]. In response to these limitations, online education, such as web-based platforms, mobile health apps, and structured e-learning programs, allows patients to learn at their convenience, in a more relaxed state, without being restricted by time or location [8]. However, these approaches often face challenges related to usability and limited interactivity, which may lead to low user engagement and reduced sustained use, thereby hindering their broader adoption [9].

In contrast, chatbots engage with users through natural and familiar conversations, enhancing their interactivity [10-12]. However, traditional chatbot systems, whether rule-based or based on conventional machine learning and natural language processing techniques, often rely on predefined scripts, limited training data, or shallow linguistic representations [13,14], making it challenging to effectively handle complex, open-ended, or personalized queries [13,15]. More recently, chatbots have increasingly been powered by large language models (LLMs), enabling more flexible, context-aware, and generative interactions [16,17], and have been explored across a range of scenarios, including mental health support [18,19] and patient education or counseling [20-22]. In the context of patient education in particular, prior studies examined the quality and understandability of responses of general LLM-powered chatbots and found that although they can provide general health-related information with reasonable quality and support patient understanding, they are limited for more specialized questions [23-26]. At the same time, current research has largely focused on evaluating system performance, including information quality and response accuracy [27,28], with limited empirical evidence on their effectiveness in practice [29], including self-management behavioral changes and workload demands on health care professionals while maintaining reliable and clinically appropriate responses.

To address these gaps, we developed an LLM-based chatbot designed and specifically trained to deliver SME by providing interactive educational content and real-time question-and-answer support, focusing on osteoporosis. Osteoporosis is a skeletal condition characterized by low bone density and impaired bone metabolism, leading to fragile bones and an increased risk of fractures [30]. Globally, osteoporosis accounts for 70% of fragility fractures, which often result in chronic pain, reduced physical activity, and significant levels of disability and dependence [31,32]. Osteoporosis can be managed proactively to prevent fractures, and even after an initial fracture, interventions can reduce the likelihood of subsequent fractures [33]. The International Osteoporosis Foundation (IOF) highlights that preventive strategies often include lifestyle modifications. Without such management, the risk of a second fracture within 2 years is notably high. However, many patients lack awareness of the importance of lifestyle changes, often relying solely on medical treatments or struggling to effectively manage the disease [34,35].

Using osteoporosis as a case study, this research aimed to explore the feasibility and preliminary effectiveness of chatbot-supported SME for chronic disease management. We conducted a formative randomized controlled trial to (1) explore its potential for gaining self-management knowledge, (2) examine its possible influence on self-management adherence, and (3) assess its potential to reduce nurses’ workload, while also evaluating the reliability of chatbot-generated responses.

## Methods

### Ethical Considerations

Prior to recruitment and data collection procedures, this study was approved by the institutional review board of the Affiliated Hospital of Guangdong Medical University (PJKT2024-216). All participants provided written informed consent before data collection and were informed that all study data would be deidentified prior to analysis. The data were stored on secure, password-protected online platforms (including BigQuery for OPBot usage data and the Wenjuanxing platform by Tencent for questionnaire data). Access to the data was restricted to authorized members of the research team to ensure participant privacy and data security, in accordance with institutional policies and applicable data protection regulations. Participants did not receive any financial compensation. However, all participants in both groups were provided with equivalent access to free follow-up services and osteoporosis-related health consultations during the study period as part of standard study support.

### Study Design and Setting

A formative randomized controlled trial was conducted in a tertiary hospital from February 2024 to March 2025.

### Participant Recruitment

The study recruited patients from February 2024 to September 2024, with follow-up completed in March 2025. Patients diagnosed with osteoporosis were recruited from the orthopedic ward of the study hospital. Eligible patients were approached by a research nurse after the diagnosis of osteoporosis had been confirmed and were invited to participate in the study during their hospitalization. Participants’ personal information was obtained from the hospital’s electronic medical record system. Recruitment took place in the hospital ward and continued until the target sample size was reached.

The inclusion criteria were (1) aged 18 years or older and (2) diagnosed with osteoporosis. The exclusion criteria were as follows: (1) unwilling or unable to use smartphones, (2) hearing impairment, (3) impaired consciousness, (4) critical illness, (5) paralyzed in bed, and (6) language barriers.

### Sample Size Justification

The sample size for this formative randomized controlled trial was determined based on methodological guidelines for pilot and feasibility studies, rather than formal hypothesis testing. The target sample size for this study was 100 participants (50 per group), determined based on practical factors, such as available resources and recruitment capacity, to support the assessment of feasibility outcomes (including retention, compliance, and acceptability) and to provide an initial estimate for future confirmatory trials.

### Randomization, Concealment, and Blinding

This study was designed as a randomized controlled trial. Researcher A assessed participants’ eligibility, obtained written informed consent, and collected baseline data. Researcher B, who was not involved in recruitment, generated the random sequence using Microsoft Excel. To ensure allocation concealment, the sequence was placed in sequentially numbered, opaque, sealed envelopes. Prior to the start of the intervention, participants were randomly assigned in a 1:1 ratio to either the intervention group or the control group according to their enrollment order and the random code indicated on the envelope. Each group included 50 participants.

Due to the nature of the intervention, blinding of participants and the intervention coordinator responsible for delivering the intervention was not feasible, as only participants in the intervention group had access to OPBot. However, to minimize potential bias, the data analyst remained blinded to group allocation during statistical analysis.

### Intervention

#### Education Chatbot: OPBot

**Figure 1:**
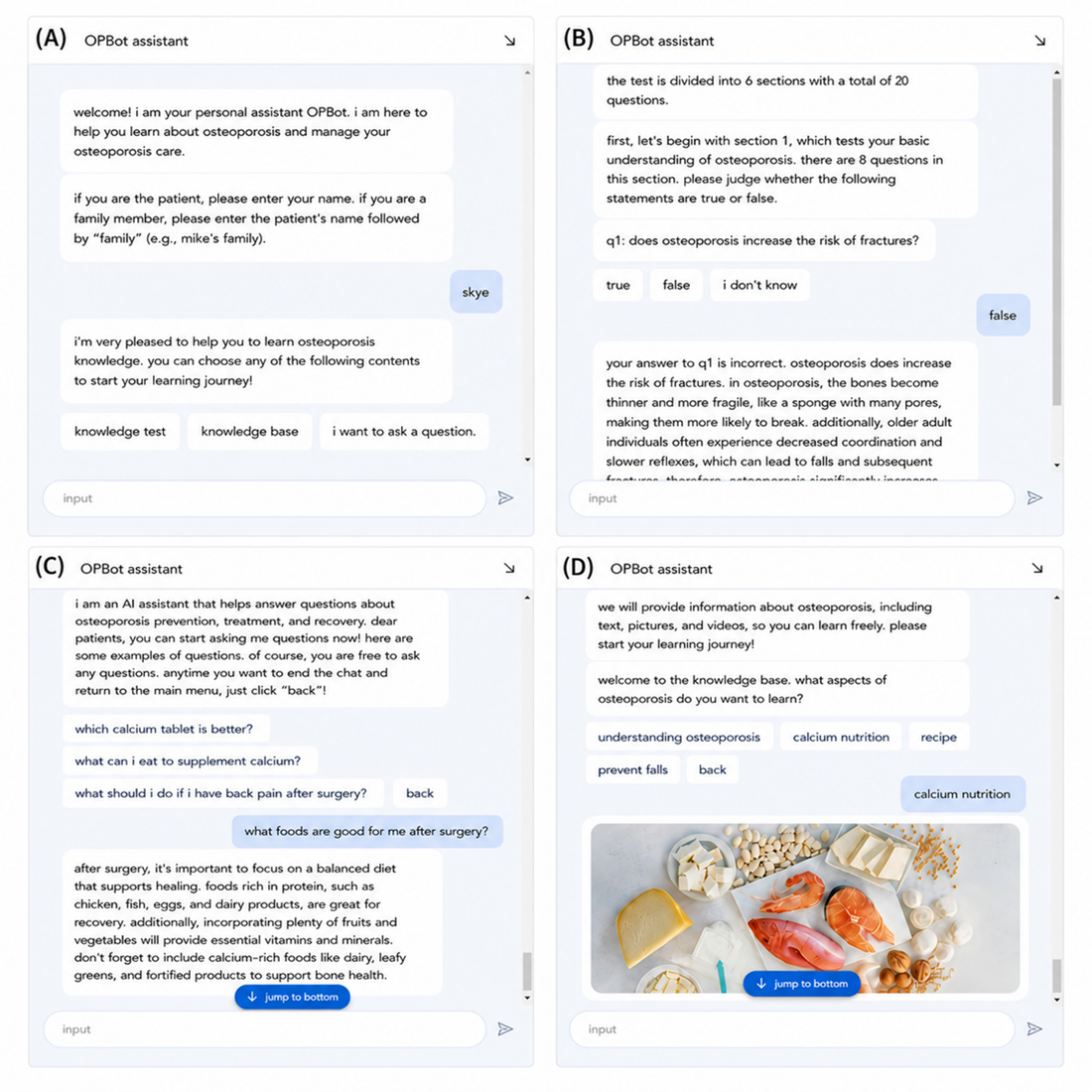
Overview of OPBot interface (translated into English for the paper). (A) Main interface, (B) knowledge assessment module, (C) question-and-answer module, and (D) knowledge base module.

OPBot is a web-based application developed collaboratively by clinical staff and computer engineers, consisting of 3 modules (Figure 1). Among them, a knowledge base module was added to deliver osteoporosis knowledge and self-management resources. Furthermore, a knowledge assessment module was introduced. This module is designed to elicit learners’ beliefs and theories, promoting the integration of new and refined concepts into their belief systems [35]. The knowledge assessment module provides educational feedback based on users’ responses, enabling the recommendation of more tailored learning content and enhancing the effectiveness of health education. This also addresses the limitation in Chinese clinical practice where time constraints often hinder pre-education assessments. It uses content adapted from the Osteoporosis Knowledge Test [36] and translated into Chinese. The user-friendly design mirrors real-world clinical assessments.

Finally, the question-and-answer module is designed to provide an efficient and convenient solution to address patients’ concerns during hospitalization and to offer ongoing support after discharge. OPBot’s training materials were sourced from the IOF and clinical consultation records. The IOF content was reviewed, translated, and adapted by orthopedic nursing experts to align with the Chinese health care context. The clinical consultation records initially included 361 entries related to osteoporosis. After removing duplicates and irrelevant content, 128 (35.5%) refined entries were ultimately selected to train OPBot. Clinical evaluation by 2 nurses (a specialized nurse and a case manager) assessed the responses generated by the question-and-answer module to ensure practical relevance. The final suitability rate was 86.7%, indicating a moderately high level of suitability. However, given the need for precision in health care, OPBot should be used with ongoing involvement from health care providers, for example, by reviewing OPBot’s responses daily and providing timely interventions when necessary. Details of the OPBot development protocol and evaluation process are provided in Multimedia Appendix 1.

#### Intervention Procedure

Participants in the intervention group learned about osteoporosis using the OPBot, a chatbot-based educational tool. The intervention was initiated during hospitalization, and participants were able to continue using the OPBot after discharge. Specifically, after osteoporosis was confirmed based on examination results (usually within 1-2 days after hospital admission), nurses informed participants of the diagnosis at their bedside and provided instructions on how to log in to and use the OPBot on their own smartphones.

During hospitalization, participants were encouraged to use OPBot as their primary source of SME. Nurses were available for consultation during working hours; however, their role was limited to addressing issues not resolved by OPBot. In addition, a specialist nurse and a case manager monitored the OPBot backend once daily to identify unresolved queries or potential risks flagged by the system. In-person intervention was provided only when necessary, based on predefined criteria (eg, unanswered questions, incorrect responses, or signs of misunderstanding), and was delivered either face-to-face or online. This targeted support was intended to supplement, rather than replace, the chatbot’s function. Before discharge, participants underwent a second knowledge assessment.

#### Comparison

The control group received traditional health education, which included face-to-face communication and written materials. After participants were enrolled, nurses provided health education and distributed an osteoporosis health handbook for participants to read. During hospitalization, participants in the control group could consult nurses during working hours regarding osteoporosis treatment and home care. All consultations were initiated by participants and conducted routinely, without systematic monitoring or proactive follow-up by nurses. No digital tools or automated support systems were used in this group. Before discharge, participants underwent a second knowledge assessment.

### Data Collection

Data were collected to assess the feasibility and to explore the potential effectiveness of the intervention.

#### Feasibility Outcomes

Feasibility was assessed from 2 aspects: nurse-reported working time and the reliability of OPBot responses. Nurse-reported working time was assessed by asking nurses to self-record their working hours during each intervention session. In the intervention group, nurses’ time included health education, consultation, and operational guidance. In the control group, nurses’ time included health education and consultation. Total working time was calculated by summing the recorded durations across sessions.

The reliability of OPBot responses was evaluated during the study. Participants asked questions and received corresponding answers through the question-and-answer module of OPBot.question-and-answer records were extracted from the OPBot backend for evaluation. Two assessors (a specialist nurse and the case manager) independently reviewed the responses. The appropriateness of each response was rated using a predefined 5-point Likert scale: “highly reliable,” “mostly reliable,” “partly reliable,” “unreliable,” and “unable to answer.” Interrater reliability between the 2 assessors was assessed using the Cohen kappa coefficient, and agreement was interpreted according to the criteria of Landis and Koch [37]. When discrepancies occurred between the 2 assessors, the responses were further reviewed by the director of the orthopedic department, and a consensus rating was reached.

#### Effectiveness Outcomes

Effectiveness was explored in terms of participants’ osteoporosis-related knowledge and self-management adherence.

Knowledge scores were assessed using the Osteoporosis Knowledge Assessment Tool (OKAT). The Chinese version of the OKAT has demonstrated good reliability and validity in previous studies [38]. The OKAT is provided in Multimedia Appendix 2. The OKAT consists of 20 items and the overall Cronbach α was 0.821. All questionnaires were administered and collected by researcher C and researcher D. Before implementation of the intervention or control group measures, the first osteoporosis knowledge score assessment was conducted as the baseline value. On the day of discharge, participants underwent a second knowledge assessment.

Self-management adherence was assessed using a self-developed questionnaire during telephone follow-ups at 1, 3, and 6 months after discharge. The questionnaire had a total score of 100 and consisted of 4 domains: calcium supplementation, consumption of calcium-rich foods, sunlight exposure, and exercise frequency. Each domain had a maximum score of 25 and was rated on a 3-point scale (0=none, 12.5=sometimes, and 25=always). The total adherence score was calculated as the sum of the scores across the 4 domains, with higher scores indicating better adherence. The items were developed based on osteoporosis self-management recommendations used in clinical practice. The full questionnaire is provided in Multimedia Appendix 3.

### Statistical Analysis

All statistical analyses were performed using R software (version 4.4.3; R Foundation for Statistical Computing). Continuous variables were summarized as mean (SD) or median and IQR, as appropriate, and compared between groups using independent *t* tests or Mann-Whitney *U* tests. Categorical variables were analyzed using chi-square tests or Fisher exact tests.

For adherence outcomes measured at 1, 3, and 6 months, mixed-effects models were used to account for within-subject correlations arising from repeated measurements over time. Depending on the nature of the outcome, logistic mixed-effects models were applied for binary outcomes, ordinal mixed-effects models for ordered categorical outcomes, and linear mixed-effects models for continuous outcomes. In all mixed-effects models, fixed effects included group (intervention vs control), time (1, 3, and 6 months), and their interaction (group×time), along with baseline covariates (sex, age, and education level). A random intercept for each participant was included to account for repeated measurements within individuals. The group×time interaction term was used to evaluate whether the effect of the intervention varied over time. To address multiplicity across adherence outcomes, calcium supplement intake was prespecified as the primary adherence outcome and was analyzed without adjustment for multiple testing. The remaining adherence outcomes, including calcium-rich food consumption, sun exposure, weekly exercise frequency, and total adherence score, were treated as secondary outcomes. Holm correction was applied to adjust for multiple comparisons among the secondary outcomes, and adjusted *P* values are reported accordingly. A 2-sided *P*<.05 was considered statistically significant.

## Results

### Study Population and Characteristics

**Figure 2:**
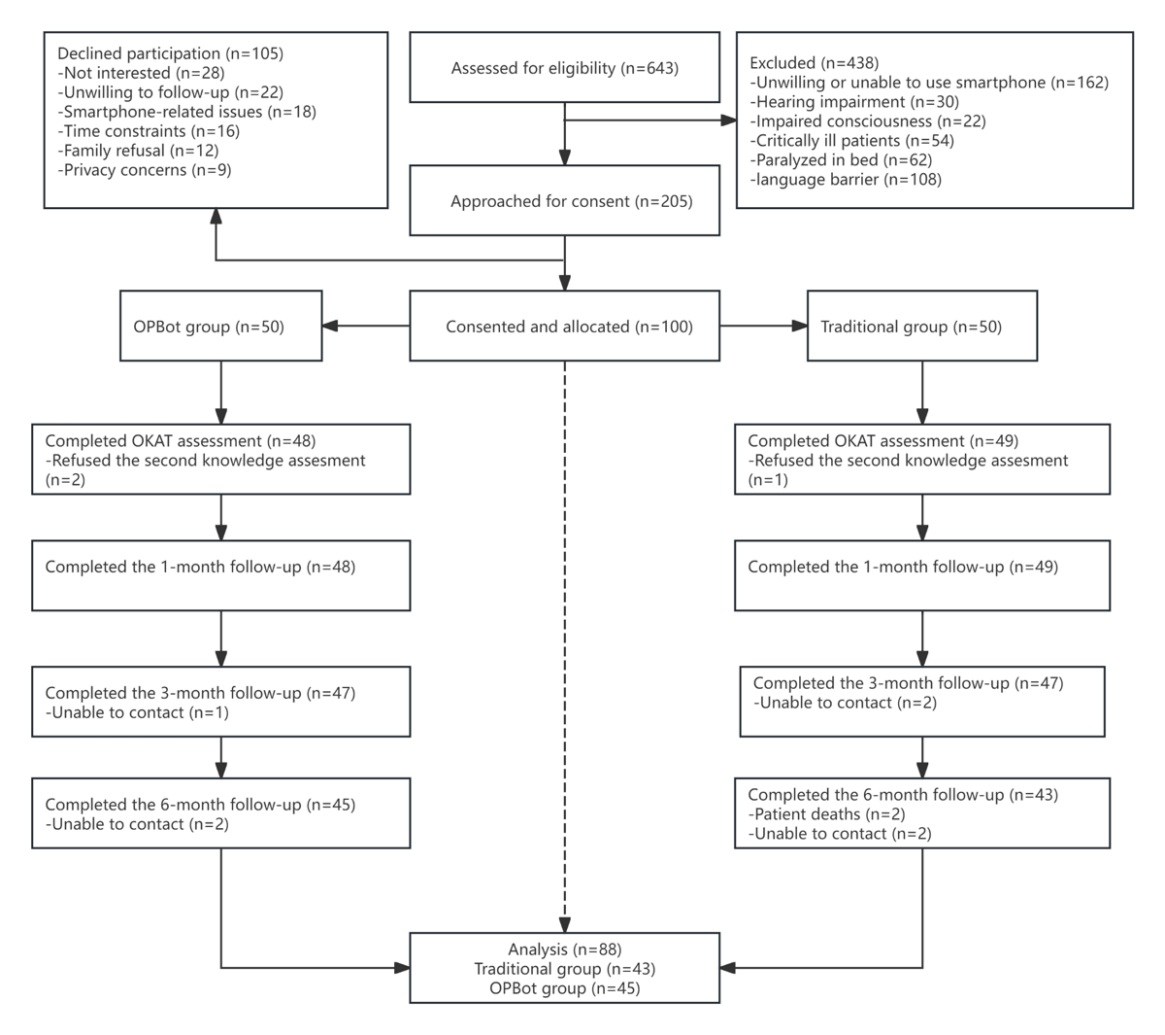
CONSORT (Consolidated Standards of Reporting Trials) flowchart of participant enrollment, allocation, and adherence. OKAT: Osteoporosis Knowledge Assessment Tool.

A total of 643 participants were screened before the study, and 15.6% (100/643) participants who met the study criteria and volunteered to participate were included and randomized into the intervention (n=50, 50%) or control (n=50, 50%) group. Ultimately, 45% (45/100) patients in the intervention group and 43 (43/100) patients in the control group completed the trial, resulting in a dropout rate of 12% (12/100). This corresponds to an overall missing data rate of 12%, as missing data arose from participant dropout. In the experimental group, 2% (2/100) participants did not complete the second knowledge assessment, and 3% (3/100) participants were lost to follow-up. In the control group, 1% (1/100) participant did not complete the second knowledge assessment, 2% (2/100) participants died, and 4% (4/100) participants were lost to follow-up (Figure 2). At baseline, no significant differences were observed in sex distribution, education level, and age between the 2 groups (Table 1). In addition, comparisons between included and excluded participants showed no statistically significant differences in baseline characteristics (Table S1 in Multimedia Appendix 4), suggesting that attrition was not associated with the measured baseline variables.

**Table 1:** Baseline characteristics of participants by study group (intervention vs control). Data are presented for participants with complete follow-up data.

		Intervention (n=45)	Control (n=43)	*P* value	
**Sex, n (%)**					.13
	Female	29 (64.4)	34 (79.1)		
	Male	16 (35.6)	9 (20.9)		
Age (years), mean (SD)		68.58 (9.4)	72.56 (9.8)	.06	
**Education, n (%)**					.84
	Illiteracy	9 (20)	7 (16.3)		
	Primary or secondary school	35 (77.8)	34 (79.1)		
	College or undergraduate	1 (2.2)	2 (4.6)		

### Feasibility Outcomes

#### Nurse-Reported Working Time

Nurse-reported working time was significantly lower in the intervention group compared with the control group (median 5.00, IQR 2.00-17.00 vs median 23.00, IQR 20.00-25.00; *P*<.001; Table 2).

**Table 2:** Knowledge scores, time interval between assessments, and nurse-reported working time by study group. Relevant findings are described in the corresponding sections.

		Intervention (n=45), median (IQR)	Control (n=43), median (IQR)	*P* value
**Knowledge score**				
	A1^a^	55.00 (42.00-70.00)	53.00 (39.50-66.50)	.66
	A2^b^	80.00 (70.00-89.00)	75.00 (65.50-80.00)	.01
Time interval between A1 and A2 (days)		5.0 (3.0-7.0)	5.0 (4.0-6.0)	.43
**Working hours**				
	Nurse-reported working time (min)	5.00 (2.00-17.00)	23.00 (20.00-25.00)	<.001

^a^A1: the baseline of knowledge score about osteoporosis.

^b^A2: postintervention osteoporosis knowledge score.

#### Reliability of OPBot

In the question-and-answer module, a total of 85 questions and corresponding responses were recorded. Initial disagreement was identified in 3 records and was resolved by consensus. Interrater agreement was almost perfect (Cohen κ=0.83). Final ratings showed that 76 (89.4%) records were highly reliable, 1 (1.2%) record was mostly reliable, 1 (1.2%) record was partially reliable, 1 (1.2%) record was unreliable, and 6 (7.1%) records were classified as unable to respond.

### Effectiveness Outcomes

#### Knowledge Score

Regarding knowledge scores, no significant difference was observed between the 2 groups at baseline (A1; median 55.0, IQR 42.0-70.0 vs median 53.0, IQR 39.5-66.5; *P*=.66). After the intervention, the intervention group demonstrated significantly higher knowledge scores (A2) compared with the control group (median 80.0, IQR 70.0-89.0 vs median 75.0, IQR 65.5-80.0; *P*=.01). The median time interval between the 2 knowledge assessments was 5.0 days in both groups (intervention: IQR 3.0-7.0; control: IQR 4.0-6.0; *P*=.43; Table 2).

#### Self-Management Adherence Outcomes

Adherence outcomes are presented in Table 3. For the primary outcome, calcium supplement intake, the main effect of group was not statistically significant (odds ratio [OR] 0.37, 95% CI 0.10-1.37; *P*=.14). However, a significant group×time interaction was observed (OR 1.49, 95% CI 1.08-2.06; *P*=.02), indicating that changes in supplement intake over time differed between the intervention and control groups. A significant main effect of time was also identified for calcium supplement intake (OR 0.75, 95% CI 0.61-0.93; *P*=.01).

For the secondary outcomes, the intervention group showed higher odds of consuming calcium-rich foods than the control group (OR 2.87, 95% CI 1.04-7.89; nominal *P*=.04). However, this association did not remain statistically significant after Holm correction for multiple comparisons. No significant group differences were observed for sun exposure, weekly exercise frequency, or total adherence scores. A significant time effect was observed for sun exposure (OR 0.97, 95% CI 0.94-0.99; *P*=.03), suggesting that sun exposure changed over time across both groups. No significant group×time interactions were identified for any secondary outcomes after Holm correction.

**Table 3:** Results of mixed-effects models for adherence outcomes over time.

			Calcium supplement intake				Calcium-rich foods consumption					Sun exposure					Weekly frequency of exercise					Total scores		
			OR^a^ (95% CI)		*P* value		OR (95% CI)		*P* value		OR (95% CI)			*P* value		OR (95% CI)			*P* value		β (95% CI)			*P* value
**Group**																								
	Control	Reference				Reference				Reference					Reference					Reference				
	Intervention	0.37 (0.10-1.37)		.14		2.87 (1.04-7.89)		.04		0.99 (0.39-2.54)			.99		1.20 (0.42-3.42)			.73		1.88 (−8.16-11.91)			.71	
**Sex**																								
	Female	Reference				Reference				Reference					Reference					Reference				
	Male	1.20 (0.52-2.77)		.66		1.46 (0.63-3.37)		.38		1.15 (0.59-2.25)			.68		0.48 (0.23-0.99)			.05		−1.06 (−8.39-6.27)			.78	
Age			0.99 (0.95-1.03)		.67		1.03 (0.99-1.07)		.18		0.97 (0.94-0.99)			.03		0.98 (0.95-1.02)			.36		−0.08 (−0.43-0.27)			.65
Education			0.94 (0.41-2.20)		.89		1.83 (0.83-4.07)		.14		0.79 (0.40-1.54)			.48		0.97 (0.44-2.13)			.94		1.54 (−5.95-9.03)			.69
Time			0.75 (0.61-0.93)		.01		1.02 (0.85-1.21)		.85		0.89 (0.76-1.05)			.18		1.05 (0.87-1.26)			.64		−1.49 (−3.29-0.31)			.11
Group×time^b^			1.49 (1.08-2.06)		.02		0.89 (0.70-1.15)		.79		1.17 (0.93-1.48)			.56		1.12 (0.85-1.48)			.79		2.25 (−0.27-4.76)			.33

^a^OR: odds ratio.

^b^Group×time: interaction between group and time.

## Discussion

### Principal Findings

This formative randomized controlled trial aimed to preliminarily explore the feasibility and potential effects of a chatbot-based SME tool (OPBot) among patients with osteoporosis. For feasibility, nurses in the intervention group spent significantly less time on SME than those in the control group, indicating that OPBot may have the potential to reduce the workload associated with patient education. In addition, the reliability evaluation of the question-and-answer module showed a high level of interrater agreement, suggesting that OPBot responses demonstrated a relatively high level of reliability in a clinical context.

For effectiveness outcomes, the intervention group had significantly higher postintervention knowledge scores compared with the control group. In this study, the time interval between baseline and postintervention assessments was relatively short (median 5 days) and did not differ significantly between groups, which may reduce the likelihood of differential bias. Nevertheless, because the OPBot intervention included a module developed based on the same knowledge assessment tool, participants in the intervention group may have been more exposed to similar content and therefore more likely to recall or recognize test items. This may have led to an overestimation of the intervention effect on knowledge outcomes.

In addition, the intervention group demonstrated a more favorable trajectory over time for calcium supplement intake, as reflected by a significant group×time interaction. This suggests that the pattern of supplement use evolved differently between groups across the follow-up period, rather than showing a consistent difference at individual time points. The intervention group also showed higher odds of consuming calcium-rich foods across time points, although this association did not remain significant after adjustment for multiple comparisons. However, no significant effects were observed for sun exposure, exercise, or total adherence scores, suggesting that the impact of the intervention on adherence was behavior specific rather than uniform across all domains. Overall, these findings indicate that chatbot-based health education may influence certain aspects of self-management adherence, although its effects may vary depending on the specific behavior.

### Comparison With Prior Work

Previous research has shown that LLM-driven chatbots can provide high-quality and easily understandable health information, demonstrating good potential in improving patient understanding and information access [23,25]. Our findings are consistent with these studies, showing that LLM chatbots may have a positive role in improving self-management knowledge.

Different adherence patterns emerged across various behaviors. Specifically, the changes in calcium supplement intake between the intervention and control groups (group×time interaction: OR 1.49, 95% CI 1.08-2.06; nominal *P*=.02), although this difference was not statistically significant after Holm correction. The intervention group consistently demonstrated higher adherence to calcium-rich foods at all time points. However, no significant differences were observed in other adherence behaviors or overall adherence scores, suggesting that the effectiveness of educational interventions is behavior specific [39]. Comparatively, behaviors such as supplement intake and dietary choices were more susceptible to the influence of chatbot-assisted education, possibly due to their easier integration into daily life, lower implementation costs, and greater consistency with existing health perceptions. For example, in some cultural contexts (including southern China), diet plays a crucial role in health management, which may further promote the adoption of relevant health recommendations. From a practical perspective, these results suggest that chatbot-based health education interventions should consider both the behavioral characteristics and cultural context of the target population when designing them. For behaviors more easily driven by information (such as diet and supplement intake), enhanced personalized recommendations and continuous feedback can improve effectiveness, while for behaviors more influenced by environment or habits (such as exercise or sun exposure), more complex behavioral support strategies may be necessary. Overall, continued access to educational support may help improve specific compliance behaviors, but its impact on overall self-management remains behavior dependent.

Moreover, although general-purpose LLM-based chatbots (eg, ChatGPT; OpenAI) have demonstrated high levels of readability and interactivity in providing general health information [23,25,28], they may still produce responses that are incomplete, insufficiently specific, or potentially inaccurate when addressing disease-specific questions, particularly in clinical contexts [23-26,40]. In contrast, the OPBot in this study was optimized for a specific disease domain, integrating a real clinical knowledge base and undergoing targeted training, enabling it to improve the professionalism and relevance of its answers while maintaining a good interactive experience. This “domain-customized” design may, to some extent, compensate for the shortcomings of general-purpose models regarding professional depth, thus making it more suitable for specific scenarios of chronic disease SME.

### Implications

Furthermore, integrating OPBot into the health care system, especially in-hospital clinical education, may help support SME delivery. Although there have been studies on LLM-based chatbots in education, there is still a lack of research on their use within hospital settings [17,25,27]. Typically, SME starts when a patient is admitted to the hospital and runs through the entire treatment and follow-up process. However, the introduction of OPBot may help reshape this traditional process. On the basis of our study, OPBot could serve as a knowledge coach, mainly responsible for providing patients with general osteoporosis knowledge and answering specialized questions. Although LLM-powered chatbots have shown great potential in personalized guidance [25,41], the accuracy and reliability of information are still key issues that must be addressed, especially in the medical field, where any misinformation may have a negative impact on patients. Therefore, current chatbots cannot completely replace the role of human providers [42]. In actual applications, neither nurses nor patients rely on OPBot alone but rather regard it as an auxiliary tool.

Furthermore, Beg [43] emphasized that artificial intelligence (AI) research should establish clear standards for ethical review and transparency to safeguard participants’ rights and mitigate potential risks. While AI shows great promise in emotion recognition, dynamic feedback, and remote intervention, it also faces challenges related to trust, ethical responsibility, and role coordination. Accordingly, this study strengthened data privacy protection, information traceability, and mechanisms for human oversight. For instance, nurses reviewed OPBot responses daily and proactively contacted patients when necessary to provide personalized guidance, reflecting the ethical principle of human-AI collaboration.

Moreover, although chatbots can provide real-time feedback and personalized learning based on patients’ needs, a lack of transparency in algorithmic logic and information sources may lead to bias or misinformation. As Beg and Verma [44] further highlighted, personalization and clinical interpretability are essential in AI-driven digital interventions. Therefore, this study implemented safety mechanisms within OPBot. For example, any inquiry related to medication or treatment automatically triggered the response “Please consult your doctor.” This design ensured that patients made medical decisions under professional supervision, embodying the importance of ethical risk control.

### Limitations

However, this study still has certain limitations. First, the reliance on self-reported adherence data is prone to recall and social desirability bias. In addition, although no significant baseline differences were observed between included and excluded participants (Table S1 in Multimedia Appendix 4), the potential impact of missing data and attrition bias cannot be completely ruled out. Second, the participants were recruited from a single tertiary hospital in China, which may limit the generalizability of the findings. Future studies should conduct multicenter, large-sample randomized controlled trials to provide higher-quality evidence. Extending the follow-up duration in future research would allow for a more comprehensive evaluation of the long-term effectiveness of chatbot-based SME, such as its impact on patients’ bone density.

### Conclusions

OPBot may have the potential to improve patients’ self-management knowledge of their disease. In health care settings with limited human resources, leveraging educational chatbots to share part of the educational workload and provide patients with basic self-management guidance may be a feasible strategy. Furthermore, to provide better personalized guidance to patients, continuously expanding the chatbot’s corpus is essential to improving its accuracy and credibility. Finally, this study offers valuable insights for SME for other chronic diseases, although further large-scale and long-term studies are needed to confirm these findings.

## Data Availability

The datasets generated and/or analyzed during this study are not publicly available due to ethical and privacy considerations but are available from the corresponding author on reasonable request.

## Appendix

Multimedia Appendix 1 The context and design process of OPBot.

Multimedia Appendix 2 Osteoporosis knowledge assessment tool (Chinese version).

Multimedia Appendix 3 Osteoporosis follow-up assessment form.

Multimedia Appendix 4 Comparison of baseline characteristics between included and excluded participants.

## Abbreviations

AI: artificial intelligence
IOF: International Osteoporosis Foundation
LLM: large language model
OKAT: Osteoporosis Knowledge Assessment Tool
OR: odds ratio
SME: self-management education
