# Supplementary material for: Preliminary Evaluation of a Large Language Model–Powered Chatbot for Osteoporosis Self-Management Education: Formative Randomized Controlled Trial
Source: JMIR Form Res. 2026 Jun 2;10:e85475. doi: 10.2196/85475 (PMC13273208; doi:10.2196/85475)
Supplement: Multimedia Appendix 1 [file formative_v10i1e85475_app1.docx]

**Appendix I**

**The Context and Design Process of OPBot**

We, a team composed of both nurses and computer scientists, decided to design a LLM-powered chatbot-based educational tool called OPBot for OP SME, leveraging the conversational interactivity of chatbots for ease of use, and fine-tuned LLMs to deliver specialised education related to OP. The clinical and computer science members worked together to design the dialogue flow and components of OPBot. Besides the commonly used question-and-answer module, we incorporated an assessment module designed to assist users in receiving educational feedback. The design was to provide users with a structured flow and functional modules to guide their use. After evaluating several chatbot framework platforms, Dialogflow CX was adopted to build the OPBot framework. We divided the entire chatbot conversation process into different flows, each serving a distinct functionality, including Default Welcome, Patient Information Collection, Knowledge Assessment, and question-and-answer. Dialogflow guides the chatbot through these flows based on the patient's responses, triggering different intents and enabling diverse functionalities. We labeled some phrases to help the chatbot understand different linguistic expressions humans use for the same entity or intent, trained the NLP model built into the Dialogflow agent, and set predefined parameters and routes, completing the framework setup.

In the Knowledge Assessment module, the items were adapted from the Osteoporosis Knowledge Assessment Test (OKAT), and translated into Chinese. We created nested flows, including test and additional education flows, to guide the user through different sections in a specific order. OPBot asks questions, extracts parameters from users’ responses, evaluates the correctness of the answers, and provides detailed explanations when necessary. Finally, the total score is calculated based on users’ responses and presented to the user. After implementing the Knowledge Assessment module, the clinical members reviewed and refined OPBot’s dialogues and responses to improve their tone and make them more closely resemble natural patient–provider conversations.

The question-and-answer module was designed to provide a faster and more accessible way for patients to resolve problems during hospitalization and to receive long-term support after discharge. We integrated Dialogflow with GPT-3, trained with two sources of data, to respond to patients' inquiries. First, we collected OP learning materials from the International Osteoporosis Foundation. The first author, an orthopedic nurse specialist, reviewed, translated, and adapted the learning materials to make them suitable for the Chinese context. For instance, exercise recommendations that are uncommon among the elderly in China (e.g., swimming and gardening) were replaced with more culturally appropriate options (e.g., Tai Chi and walking). We created new recipes based on local dietary preferences, highlighting foods with high calcium content. The materials were further revised, and overly technical terms were replaced to improve readability and patient comprehension. Moreover, we added information to explain ``why'' behind the recommended activities. For example, patients were advised to sunbathe between 8:00 and 9:00 AM during summer, as this timing promotes vitamin D synthesis while minimizing the risk of sunburn. Secondly, to make the chatbot conversations more natural and patient-centered, we collected 361 anonymized consultation messages between the case manager and patients regarding OP, recorded between January and October 2023 to train OPBot, after removing duplicate and redundant entries, resulted in 128 unique records.

Following the completion of the Assessment and question-and-answer modules, we recruited patients to test the OPBot. During the testing, minimal usage was observed in the absence of provider intervention. NR1 and NR2 reported that some patients wanted providers to demonstrate how to ask questions or what types of questions they could ask. It was clear that while patients could finish the assessment with the providers' help, they did not know where to start asking questions. To better support patients, sample questions were added to the question-and-answer module as guidance, such as “What foods are good sources of calcium?” Yet, there was little improvement in terms of encouraging patients to use it more. In the end, a Knowledge Base module was introduced to support patients in developing a deeper understanding of OP. This module offers systematic and comprehensive information on OP and self-management. Carefully selected educational materials were converted into conversational scripts to structure the dialogue flow. Images and videos were embedded to enrich the learning experience.

**The Resulted of OPBot**

An overview of OPBot is shown in Figure 1. When users enter OPBot, it proactively welcomes them and introduces itself. On the welcome page, users are required to enter their names so that the case manager can track interactions and ensure the safe use of the question-and-answer module. The system then records basic user information, including the user’s name, to manage login records, facilitate timely follow-up, and ensure data security. OPBot then presents functional options as selection buttons, including Knowledge Assessment, Knowledge Base, and question-and-answer, allowing users to choose according to their needs.

In the Knowledge Assessment module, the chatbot engages users by asking questions to evaluate their understanding of OP and its management. Guided by OPBot, users complete a test consisting of 20 questions divided into 6 sections. During the test, OPBot assesses the correctness of user responses and provides targeted education based on their answers to correct any misconceptions. After each section, OPBot identifies specific areas of weakness for further educational intervention. If users remain confused, they can access the question-and-answer module at any time to ask their questions. Once they finish their inquiries, they can return to the assessment module to continue with the next section. Upon completing all sections, users receive their final assessment score.

When users enter the question-and-answer module, OPBot introduces the feature and provides guidance on how to formulate questions. Users can freely ask questions and receive answers. Additionally, OPBot lists frequently asked questions as selectable buttons, offering examples to help users frame their inquiries. Users can click these buttons to quickly access answers to common questions.

The Knowledge Base module opens with a main menu featuring four key topics: Understanding OP, Calcium Nutrition, Recipes, and Fall Prevention. After selecting a topic, users proceed to a secondary menu where they choose specific learning materials aligned with their needs. When users select specific content, OPBot engages them in a conversational format, guiding them step-by-step through the learning process about OP. Notably, OPBot keeps users logged in for approximately 10 minutes after inactivity. If users return within this period, the interaction is treated as the same session to avoid repeated logins. This design supports smoother and more continuous user interaction.

**Internal Validation Process**

To ensure the suitability of OPBot in clinical practice, a specialist nurse and a case manager who participated in the co-design process evaluated the quality of its responses.

30 questions were randomly selected from a pre-designed question bank. Both assessors independently submitted the same set of 30 questions to OPBot via the question-and-answer module, yielding 60 responses in total (30 questions × 2 assessors).

The assessors then rated the appropriateness of each response using a predefined five-point Likert scale: “highly reliable,” “mostly reliable,” “partly reliable,” “unreliable,” and “unable to answer” (in which case OPBot would indicate that the question was beyond its capacity and recommend consulting clinicians). To assess consistency between the two raters, we calculated the inter-rater agreement and Cohen’s kappa coefficient. Of the 60 questions, identical ratings were assigned to 57, with discrepancies observed in 3 cases. The kappa coefficient was 0.95, indicating excellent inter-rater reliability.

For the 3 questions with rating discrepancies, we invited the director of the orthopedic department to review them. Through discussion, a consensus rating was reached to resolve discrepancies and finalize the evaluation. The combined evaluation included 60 responses, of which 52 were rated as “highly reliable,” 1 as “mostly reliable,” 6 as “partly reliable,” and 1 as “unreliable.” Accordingly, 86.7% of OPBot’s responses were rated as highly reliable.

Although OPBot demonstrated a high level of response reliability, clinical practice requires stringent accuracy standards. Therefore, in our study, regularly monitoring OPBot’s responses by clinicians is needed.
