# Supplementary material for: Preliminary Evaluation of a Large Language Model–Powered Chatbot for Osteoporosis Self-Management Education: Formative Randomized Controlled Trial
Source: JMIR Form Res. 2026 Jun 2;10:e85475. doi: 10.2196/85475 (PMC13273208; doi:10.2196/85475)
Supplement: Multimedia Appendix 2 [file formative_v10i1e85475_app2.docx]

**Appendix II**

**中文版骨质疏松知识评价工具**

Osteoporosis Knowledge Assessment Tool (Chinese Version)

1. 骨质疏松症导致骨折的风险增加。 £是 £否

Osteoporosis leads to an increased risk of bone fractures. □YES □NO

1. 骨折发生前骨质疏松症通常会引起的症状（如疼痛）。 £是 £否

Osteoporosis usually causes symptoms (such as pain) before a fracture occurs. £YES £NO

1. 在童年结束时具有较高的骨峰值，未采取有效措施，在以后的生活中会引起骨质疏松症。

£是 £否

Having a high peak bone mass at the end of childhood, without taking effective measures, will lead to osteoporosis later in life. £YES £NO

1. 男性患骨质疏松较常见。 £是 £否

Osteoporosis is more common in men. £YES £NO

1. 吸烟可导致骨质疏松症。 £是 £否

Smoking can lead to osteoporosis. £YES £NO

1. 与其他种族相比，白人妇女患骨质疏松的风险最高。 £是 £否

Compared to other races, white women have the highest risk of developing osteoporosis.

£YES £NO

1. 跌倒会引起骨折。 £是 £否

Falls can cause bone fractures. £YES £NO

1. 80 岁时，大多数女性都会患骨质疏松症。 £是 £否

By the age of 80, most women will have osteoporosis. £YES £NO

1. 从 50 岁算起，大多数女性在去世前会患有骨质疏松症。 £是 £否

From age 50 onwards, most women will have osteoporosis before they die. £YES £NO

1. 任何类型的身体活动都有益于骨质疏松症。 £是 £否

Any type of physical activity is beneficial for osteoporosis. £YES £NO

1. 在临床危险因素中会被告诉是否存在骨质疏松症危险。 £是 £否

Clinical risk factors can tell you if you are at risk for osteoporosis. £YES £NO

1. 有骨质疏松症家族史的更易患骨质疏松症。 £是 £否

People with a family history of osteoporosis are more likely to develop it. £YES £NO

1. 每天 2 杯奶可以补充足够的钙。 £是 £否

Drinking 2 glasses of milk a day can provide enough calcium. £YES £NO

1. 西兰花和沙丁鱼是钙的良好来源（无法摄取奶制品的人）。 £是 £否

Broccoli and sardines are good sources of calcium (for people who cannot consume dairy products). £YES £NO

1. 补钙可防止骨质疏松症。 £是 £否

Taking calcium supplements can prevent osteoporosis. £YES £NO

1. 适度饮酒对骨质疏松影响不大。 £是 £否

Moderate alcohol consumption has little effect on osteoporosis. £YES £NO

1. 高盐摄入是骨质疏松症的危险因素。 £是 £否

High salt intake is a risk factor for osteoporosis. £YES £NO

1. 绝经后 10 年，有一个小的骨丢失量。 £是 £否

In the 10 years after menopause, there is a small amount of bone loss. £YES £NO

1. 绝经后的任何年龄，使用激素治疗可防止进一步的骨质丢失。 £是 £否

At any age after menopause, using hormone therapy can prevent further bone loss. £YES £NO

1. 在中国，骨质疏松症无有效治疗。 £是 £否

In China, there is no effective treatment for osteoporosis. £YES £NO
