# Supplementary material for: Preliminary Evaluation of a Large Language Model–Powered Chatbot for Osteoporosis Self-Management Education: Formative Randomized Controlled Trial
Source: JMIR Form Res. 2026 Jun 2;10:e85475. doi: 10.2196/85475 (PMC13273208; doi:10.2196/85475)
Supplement: Multimedia Appendix 3 [file formative_v10i1e85475_app3.docx]

**Appendix III**

**骨质疏松随访评估表**

**Osteoporosis Follow-up Assessment Form**

1. 是否知道自己有骨质疏松症 □是 □否

Knowledge of osteoporosis diagnosis £YES £NO

1. 随访时间 □出院后一个月 □出院后三个月 □出院后六个月

Follow-up Time £1 month after discharge £2 month after discharge £3 month after discharge

1. 现存问题 □疼痛 □其他 __________ □不适用

Existing Problems □Pain □Others □Not applicable

1. 手术切口 □愈合 □结痂 □渗液 □渗血

Surgical incision □healing □crusting □drainage □bleeding

10. 支具佩戴 □坚持佩戴 □偶尔佩戴 □几乎不佩戴 □不需要佩戴

Brace wearing status □Regular □Occasional □Rarely □No need

1. 是否服用钙片补充剂 □是 □否 _________________（原因）

Calcium supplement intake £YES £NO_______________（Reason）

1. 是否摄入含钙食物 □经常 □偶尔 □从不 _______（原因） □不知道什么是高钙食物

High-Calcium Food Intake

£Regular £Occasional £No intake________（Reason） £Unfamiliar with high‑calcium foods

13. 日晒情况（平均每日） □≤30min □30-60min □≥1h □室外务工 □几乎不 ______（原因）

Sun exposure duration （Average daily）

□≤30min □30-60min □≥1h □Outdoor work □Rarely_____________（Reason）

14. 运动 □床上活动 □散步 □养生操 □广场舞 □从不 □其他_________ □运动时长 _______

Exercise

□Bed activities □Walking □Health Qigong □Group recreational dancing □Not at all □Others______ □Exercise duration________

15. 每周运动频率 □每周≥3次 □每周1-2次 □偶尔 □从不 _______（原因）

Weekly exercise frequency □≥ 3 times □1-2times □Occasional □Not at all______（Reason）
