# Supplementary material for: Preliminary Evaluation of a Large Language Model–Powered Chatbot for Osteoporosis Self-Management Education: Formative Randomized Controlled Trial
Source: JMIR Form Res. 2026 Jun 2;10:e85475. doi: 10.2196/85475 (PMC13273208; doi:10.2196/85475)
Supplement: Multimedia Appendix 4 [file formative_v10i1e85475_app4.docx]

**Table S1**

**Table S1:** Comparison of baseline characteristics between included and excluded participants.

|  | Included participants (n=88) | Excluded participants (n=12) | P |
| --- | --- | --- | --- |
| Group |  |  | 0.538 |
| Control | 43 (48.86%) | 7 (58.33%) |  |
| Intervention | 45 (51.14%) | 5 (41.67%) |  |
| Sex |  |  | 0.989 |
| Female | 63 (71.59%) | 8 (66.67%) |  |
| Male | 25 (28.41%) | 4 (33.33%) |  |
| Age | 70.00(65.00-78.00) | 62.00 (58.75-74.25) | 0.075 |
| Education |  |  | 0.367 |
| Illiteracy | 16 (18.18%) | 3 (25.00%) |  |
| Primary school/Secondary school | 69 (78.41%) | 8 (66.67%) |  |
| College/Undergraduate | 3 (3.41%) | 1 (8.33%) |  |
